# Abdominal Wall Endometrioma: A Diagnostic Enigma—A Case Report and Review of the Literature

**DOI:** 10.1155/2019/6831545

**Published:** 2019-03-26

**Authors:** Ketan Vagholkar, Suvarna Vagholkar

**Affiliations:** Department of Surgery, D. Y. Patil University School of Medicine, Navi Mumbai 400706, MS, India

## Abstract

**Background:**

Abdominal wall endometriomas are quite uncommon. They are usually misdiagnosed by both the surgeon and the gynaecologist. Awareness of the details of this rare condition is therefore essential for prompt diagnosis and adequate treatment.

**Introduction:**

Endometriosis though a condition commonly seen in the pelvic region can also occur at extrapelvic sites giving rise to a diagnostic dilemma. Abdominal wall endometrioma is one such complex variant of extrapelvic endometriosis with an incidence of less than 2% following gynaecologic operations.

**Case Report:**

A case of abdominal wall endometrioma diagnosed clinically and treated by wide surgical resection is presented to highlight the importance of clinical evaluation in the diagnosis of this condition.

**Discussion:**

The etiopathogenesis, presentation, investigations, and management are discussed briefly.

**Conclusion:**

Clinical evaluation confirmed by supportive imaging is diagnostic. Wide local excision is the mainstay of treatment.

## 1. Introduction

Endometriosis is defined as a benign inflammatory disease characterized by the presence of ectopic endometrial tissue which is oestrogen dependent. When the lesion is a well circumscribed mass it is designated as an endometrioma. Abdominal wall endometrioma (AWE) is an uncommon aftermath of gynaecologic operations such as caesarean section or an abdominal hysterectomy. The incidence varies from 1 to 2% [1]. The diagnosis is elusive causing intense pain and discomfort to the patient. Awareness of this entity can help the surgeon to make an early diagnosis and deliver prompt surgical treatment. A case of abdominal wall endometrioma is presented along with a brief review of literature.

## 2. Case Report

A 29-year-old female presented with a mass on the anterior abdominal wall present for 1 year. The mass had gradually increased in size over this period of time. She complained of continuous discomfort and pain which became worse during menses. The mass increased in size during menses as per the patient's description. She had undergone a caesarean section six years back. There was no other significant history. Her menses were regular with no bladder or bowel disturbances.

**Figure 1:**
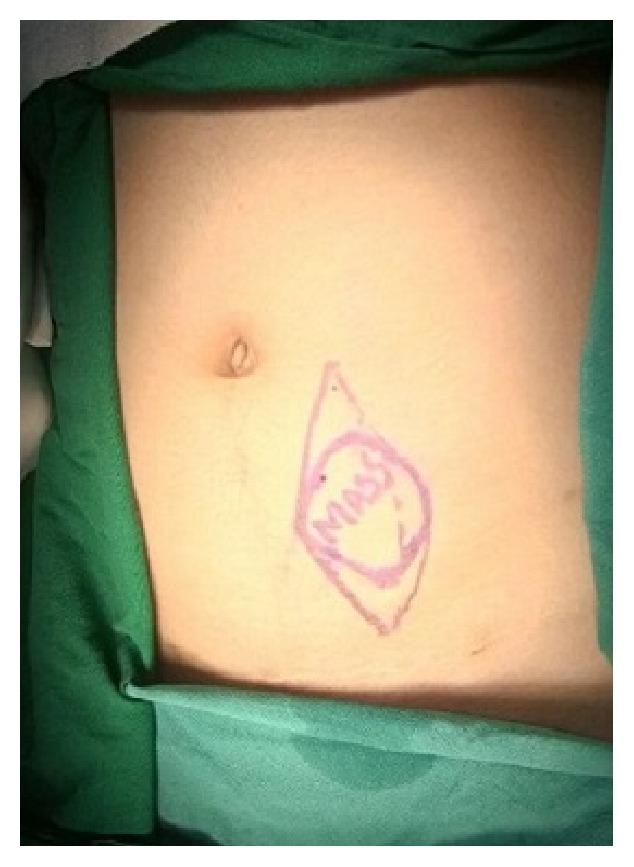
Palpable mass in the infraumbilical region to the left of the midline.

Physical examination of the abdomen revealed a circumscribed mass measuring approximately 6 cms in diameter in the infraumbilical region to the left of the midline (Figure 1).

The mobility of the swelling became restricted on contracting the underlying muscles suggestive of infiltration of the underlying musculoaponeurotic structures.

**Figure 2:**
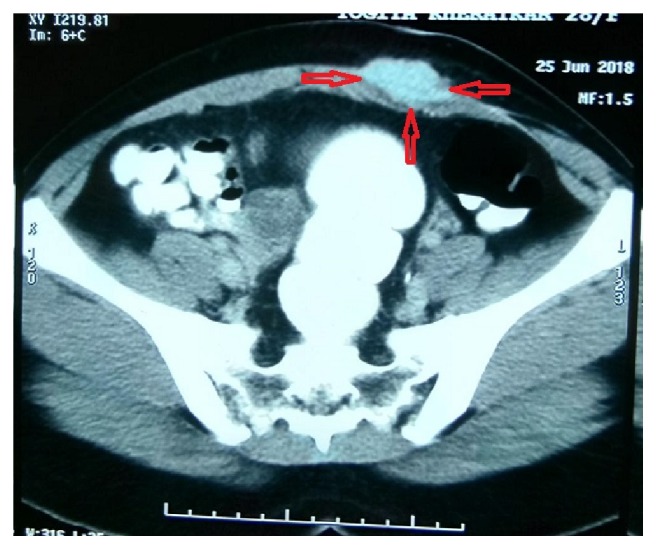
CECT showing enhancing mass in the subcutaneous tissues infiltrating the deeper musculoaponeurotic structures.

Laboratory investigations were within normal limits. A contrast enhanced CT scan was done during her menses. The CT scan revealed a contrast enhancing lesion in the subcutaneous tissues infiltrating the underlying musculoaponeurotic structures highly suggestive of an abdominal wall endometrioma (Figure 2).

**Figure 3:**
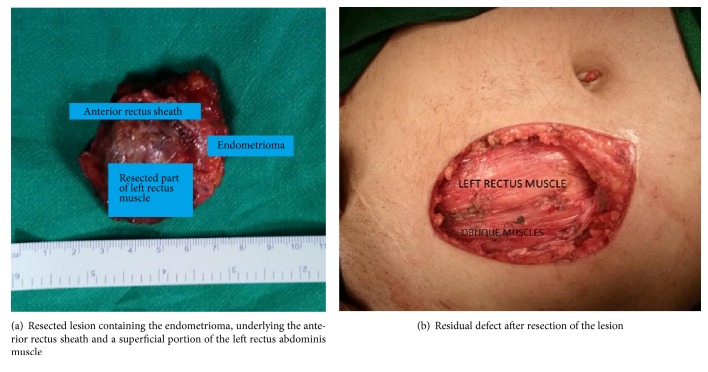


She underwent surgical resection. The endometrioma was resected along with the portion of the underlying aponeurosis and rectus abdominis muscle (Figures 3(a) and 3(b)).

**Figure 4:**
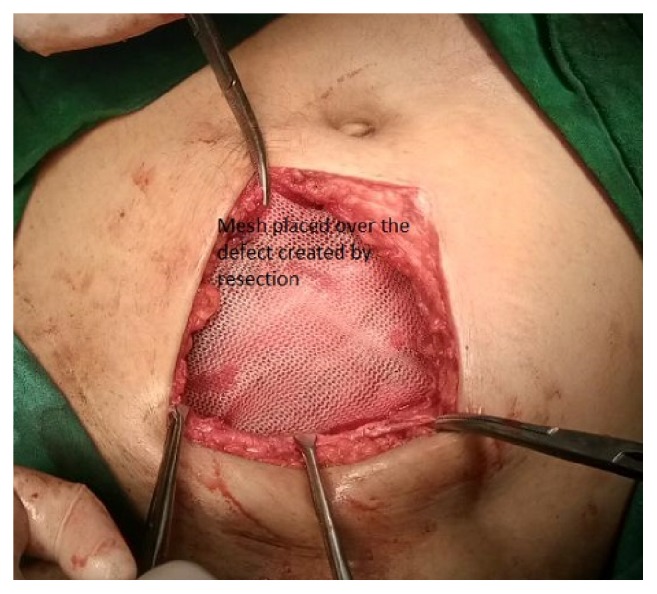
Mesh reconstruction of the defect.

**Figure 5:**
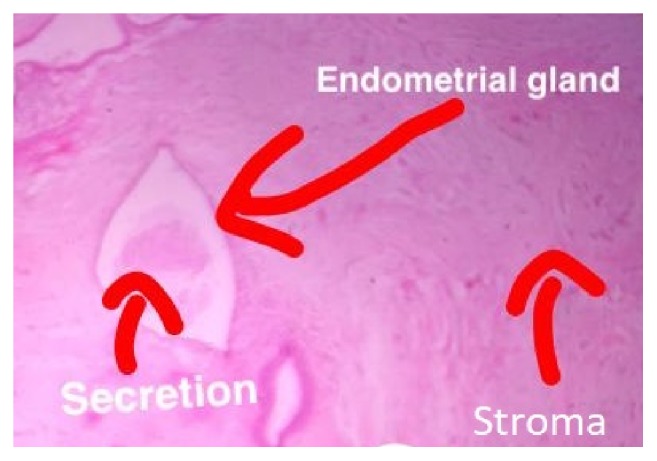
Histopathology of the specimen showing endometrial glands and stroma (H&E staining, magnification 10x).

A polypropylene mesh was placed over the defect created by the resection and fixed all around the defect to the anterior rectus sheath with nonabsorbable sutures (Figure 4). The postoperative course of the patient was uneventful. The histopathological evaluation of the resected specimen revealed endometrial glands and stroma with clear resection margins (Figure 5).

Following the procedure there was no seroma at the operative site and the patient noted complete relief of symptoms.

## 3. Discussion

The presence of endometrial glandular and stromal tissue outside the uterus is called endometriosis. It is seen in women of active reproductive age [1, 2]. The common sites for endometriosis are the ovaries, pelvis, lower intestinal tract which includes the sigmoid colon, and urinary system especially the bladder. Scar endometriosis is another evolving entity. Bits of endometrial tissue get seeded into the incision at the time of surgery giving rise to endometriotic deposits. The most common operations which can lead to this are hysterectomy and caesarean section.

Various theories have been proposed to explain the etiopathogenesis of abdominal wall endometriomas [3]. The transport theory explains that direct inoculation or transport of the endometrial tissue into surgical scars or adjacent tissues during surgery is responsible for abdominal wall endometriosis. The metaplastic theory proposes that primitive pleuropotential mesenchymal cells that have undergone differentiation and metaplasia may lead to the development of abdominal wall endometrioma.

Previous caesarean section or hysterectomy, high parity, and increased menstrual flow are known risk factors for AWE. The Esquivel triad comprised of a palpable tumour, cyclic pain, and a history of lower caesarean section is virtually diagnostic of AWE [3].

However this may not be the presentation in all cases. Subtle variation in clinical features may be seen. Hence an elaborate history with respect to time frames of surgical events and commencement of symptoms is extremely important. Usually the time interval between the index surgery and onset of symptoms ranges between three and six years. In the case presented it was almost six years. The diagnostic clue is exacerbation of local symptoms of severe pain and discomfort accompanied by expansion of the mass with the onset of menstruation. This was typically seen in the case presented thus enabling an accurate clinical diagnosis.

A variety of imaging modalities are available to confirm the diagnosis of AWE. The surgeon has to be aware of the findings revealed by each modality in order to avoid misdiagnosis. Doppler ultrasound typically reveals a solid hypoechoic lesion containing internal vascularity. It has a sensitivity of 92% [4–6]. Contrast enhanced computed tomography (CECT) done during menses can be diagnostic as seen in the case presented. The exact location, size, and nature of the mass based on contrast enhancement are diagnostic [7]. MRI has better contrast resolution than CECT and ultrasound [8]. It can detect smaller lesions and will also identify haemorrhage associated with the endometrial lesion. In addition it also helps in the delineation between muscle and subcutaneous tissue as well as infiltration of deeper structures.

Fine needle aspiration cytology (FNAC) runs the risk of needle track implantation of the endometriotic lesion. FNAC will show endometrial-like epithelial cells, stromal cells, and hemosiderin laden macrophages. It is extremely difficult to diagnose scar endometriosis by FNAC [9].

Histopathological evaluation of the resected mass is confirmatory. Any two out of the three classical features are diagnostic. These include endometrial glands, endometrial stroma, and hemosiderin laden macrophages.

The risk of developing a clear cell carcinoma in these lesions is less than 1%. Advanced age, postmenopausal state, and tumour size greater than 9 cms are risk factors for malignant transformation. The five-year survival rate in such cases is 80%.

The differential diagnosis may include a variety of conditions such as hernia, lipoma, desmoid tumour, or primary or metastatic malignancy. Therefore a careful history with proper interpretation of radiological findings can help in making a correct preoperative diagnosis.

Various pharmacologic treatments have been used for AWE [10, 11]. These include oral contraceptive pills containing progesterone, antioestrogens such as danazol, and gonadotropic agonists like leuprolide acetate [11]. Unfortunately the success rate with these medications is extremely poor, thus necessitating surgery in the majority of cases [12].

Surgery consists of wide local excision with an aim of achieving a clear resection margin [13]. The underlying musculoaponeurotic structures involved must be resected. This may lead to a wider defect. A mesh reconstruction of the defect is therefore necessary in a majority of cases as was done in the case presented. The indicators of an incomplete resection are the development of seromas at the operative site and an early onset of the same pain in the postoperative period as was experienced at the initial presentation [13, 14]. Awareness of the AWE can help in developing preventive strategies during the course of the index surgery. Rigorous high jet saline irrigation of the wound edges prior to closure can eliminate the condition [15].

## 4. Conclusion

Abdominal wall endometrioma is a rare entity developing after gynaecologic surgery. Awareness of this condition is essential for making a diagnosis. A proper history and physical examination during menses can help in arriving at a diagnosis. CECT will confirm the diagnosis. Wide local excision is the mainstay of treatment. High pressure saline irrigation of the wound edges can prevent the development of AWE at the time of the index surgery.
